# Mapping vulnerability to climate-related hazards to inform local authority action in adaptation: A feasibility study

**DOI:** 10.1016/j.puhip.2024.100549

**Published:** 2024-09-21

**Authors:** J. Howkins, Daniela N. Schmidt, James Thomas, Robert Hayward, Y.T. Eunice Lo, Jeffrey Neal, James Lewis, Elspeth Carruthers, Samuel Coleborn, Virginia Murray, Isabel Oliver

**Affiliations:** a UK Health Security Agency, London, UK; b Bristol Medical School, University of Bristol, UK; c School of Earth Science, University of Bristol, UK; d Jean Golding Institute, University of Bristol, UK; e Department for People, South Gloucestershire Council, UK; f Cabot Institute for the Environment and Elizabeth Blackwell Institute for Health Research, University of Bristol, UK; g School of Geographical Sciences, University of Bristol, UK; h Fathom, Clifton Heights, Triangle West, Clifton, Bristol, UK; i Data Analytics & Surveillance, UK Health Security Agency, London, UK; j Whittington Hospital, London, UK; k NIHR Health Protection Research Unit in Evaluation of Interventions, University of Bristol Medical School, School of Population Health Sciences, Bristol, UK

## Abstract

**Background:**

Local authorities have a crucial role in building community resilience to the health effects of a changing climate. Support in achieving local action can be provided through improving available public health intelligence to inform decision making. We aimed to co-develop with a local authority a tool mapping vulnerability to climate related hazards.

**Methods:**

We conducted a feasibility study, exploring through stakeholder engagement local priorities and levers for action in adaptation that could be informed by provision of increased intelligence. This informed co-development of a proof-of-concept tool.

**Results:**

Stakeholders reported needs in better understanding the intersection between vulnerability and hazard to facilitate partnership working, decision making, and targeting of interventions. We developed a mapping tool, using nationally available data, overlaying a vulnerability index with hazard (heat and flooding) exposure.

**Conclusions:**

Mapping tools are feasible methods by which public health intelligence to support climate change adaptation planning can be shared. Barriers to action may result from the complexity of vulnerability, concerns of unintended consequences, and resource constraints. Co-development with local expertise is necessary to ensure that outputs add value to local response. This tool will now be piloted to gather feedback on useability, usefulness, and potential improvements.

## What this study adds

We create a novel climate vulnerability mapping tool designed in partnership with Local Authority, integrating understanding of their needs and potential levers for action from the outset.

We add value to currently available tools through this co-development, and by processing vulnerability and hazard data to show the overlap of risk to support understanding of this complex picture.

## Implications for policy and practice

Provision of improved public health intelligence is vital in creating a more enabling environment to community resilience building.

Current evidence shows that even when local actors are consulted in the development of climate mapping tools for their use, they rarely have impact.

By co-designing then subsequently trialling our intervention, we hope to garner greater detail on what works, and how to improve future impact.

## 1 Background

Climate change is impacting mortality and morbidity of people across the world [1]. Key health risks in Europe are heat, the impact of heat and drought on nutritional quality, flooding, and water shortages [2]. In the United Kingdom (UK), the heatwaves in 2022 were linked to 3,271 deaths above the five-year average [3]. Flooding incidents can pose acute risks to life and are associated with a major impact on mental health and wellbeing [4], which can persist for years after the event [5]. Risk from heatwaves and floods are rated as very high in the UK Climate Change Risk Assessment (CCRA3) and are projected to increase [6].

Some groups of the population are known to be more likely to suffer adverse health outcomes when exposed to heat, flooding, and other climate-related hazards. Older people and women are more vulnerable to adverse heat-related health outcomes in England and Wales [7], and urban residents are generally more exposed to heat due to the urban heat island effect [8]. Inclusion health groups [9] such as homeless populations are more vulnerable to the outcomes of extreme weather [10]. Higher population density, level of economic inequality (as measured by the Gini index), and lower access to green space are also associated with increased heat-mortality risk [11]. UK population groups vulnerable to health impacts of flooding are similar, with greatest impact on younger and older age-groups, and those with pre-existing physical or mental ill health [12]. Climate change may also itself be a driver of increasing health inequalities [13]. Adaptation efforts are failing to keep pace with the increasing risk to health [14], widening the adaptation gap. Local Authorities (LAs) in the UK have a key role in strategic planning and in commissioning local services, including in response to the climate emergency. However, interventions with long time-horizons for benefit, such as in climate change adaptation, can be challenging to prioritise given limited time and resources and competing priorities. Efforts to help communities become more resilient are vital in reducing vulnerability to climate change in the UK [15] with local expertise enabling effective translation of national policies [16].

In the context of increasing exposure to hazard, more support to LAs is required, including in building technical capacity [17]. Tools have been developed to support decision making, but present attempts to assess vulnerability have so far been shown to rarely influence effective action [18]. Better cross-system collaboration and co-development of such tools may enable better appreciation of the complexity of interactions between hazard and vulnerability, whilst maximising focus on what can practically be influenced at LA level [19]. We conducted an explorative feasibility study with one LA, aiming to understand the needs at the local level and how a mapping tool might support decision making in building long term resilience. We used the insights gained to build a mapping tool that would subsequently be trialled by the LA to see how it might in fact be used, and where future iterations might require further work. This paper presents the first stages of co-development with the LA in understanding needs, levers for action, and building the mapping tool. South Gloucestershire Council was chosen as a LA in Southwest England that has been active in climate adaptation after declaring a climate emergency in 2019. The Southwest of England has the largest projected change in combined heat hazards (incorporating daily maximum and minimum temperatures, temperature variability and vapour pressure) in the UK [20].

## 2 Methods

**Fig. 1:**
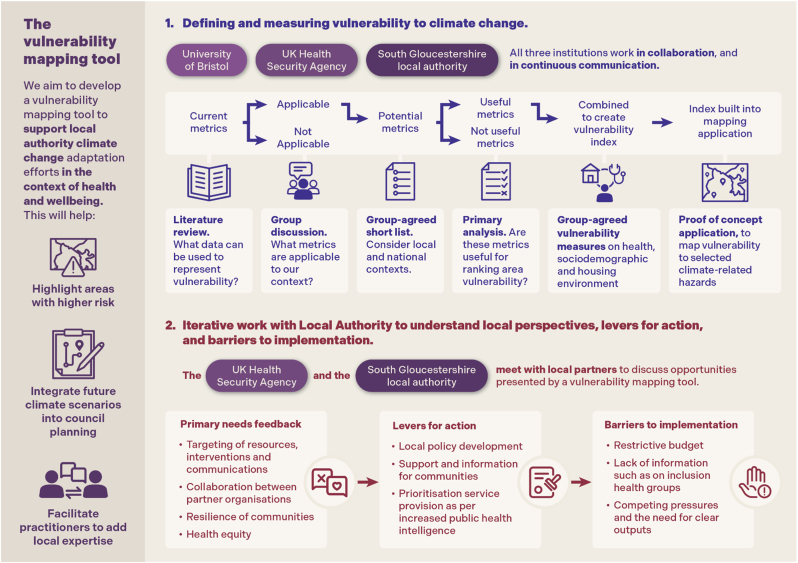
Method of engagement of the working group and co-development of the project.

This feasibility study comprised of assessing LA stakeholder requirements for the applicability of a mapping tool, then development of a proof-of-concept (POC) mapping application, due later for subsequent trial with the same LA. To assess need and applicability, we conducted 1-to-1 discussions with 12 key SGC and local partnership stakeholders, four small group workshops and presentations with SGC staff, and reviewed SGC climate-related policy and strategy documents for additional context. Stakeholders were accessed through convenience and purposive sampling [stakeholder engagement list available in supplementary material] and represented a single, LA-focused consideration on what information might be useful for them in a high-level map of vulnerability to climate hazards. Results of this engagement are presented grouped into themes identified by stakeholders as key, and through potential use cases described. Gathered evidence informed co-development of the POC mapping application [Fig. 1].

Vulnerability was conceptualised as per the Intergovernmental Panel on Climate Change [2]. Data sources were limited to nationally available, non-patient identifiable information to allow future expansion of the tool to the rest of England, and for reliability of data in line with similar studies [21]. Selection of metrics included in the index followed the methods laid out in section 1 of Fig. 1: the working group discussed metrics evidenced in the literature [Supplementary Material Table 3], selected those applicable to the geographical context, and created a shortlist [Table 1]. The code for analysis for inclusion into the index is provided [data availability statement]. Vulnerability measurement through index aggregation followed the hierarchical methodology described by Tate [22] and used in the English Indices of Multiple Deprivation (IMD) [23] [supplementary material]. Included metrics and domain groupings are shown in Table 1.

**Table 1:** Metrics used to create an index of vulnerability (data from UK 2021 census results unless otherwise noted as from 2019 IMD) [23,32].

Metrics included in vulnerability measurement		Grouping result from primary analysis	
Age	<5 years old	Sensitivity domain	Younger people sub-domain
	>65 years old		Older people sub-domain
Ethnicity	Main language is not English (English or Welsh in Wales): Cannot speak English well	Adaptive capacity domain	Language sub-domain
	Main language is not English (English or Welsh in Wales): Cannot speak English		
Education	No qualifications		Income sub-domain
	Level 1		
	Level 2 qualifications		
Income – 2019 IMD income deprivation	Adults and children in Income Support families		
	Adults and children in income-based Jobseeker's Allowance families		
	Adults and children in income-based Employment and Support Allowance families		
	Adults and children in Pension Credit (Guarantee) families		
	Adults and children in Universal Credit families where no adult is classed within the ‘Working - no requirements' conditionality group		
	Adults and children in Working Tax Credit and Child Tax Credit families not already counted, that is those who are not in receipt of Income Support, income-based Jobseeker's Allowance, income-based Employment and Support Allowance, Pension Credit (Guarantee), and whose equivalised income (excluding housing benefit) is below 60 per cent of the median before housing costs		
	Asylum seekers in England in receipt of subsistence support, accommodation support, or both.		
Unpaid carers	Provides 9 h or less unpaid care a week		Helping others sub-domain
	Provides 10–19 h unpaid care a week		
	Provides 20–34 h unpaid care a week		
	Provides 35–49 h unpaid care a week		
	Provides 50 or more hours unpaid care a week		
General Health	Bad health	Health domain	
	Very bad health		
Disability	Disabled under the Equality Act: Day-to-day activities limited a lot		
	Disabled under the Equality Act: Day-to-day activities limited a little		
	Disability: Not disabled under the Equality Act: Has long term physical or mental health condition but day-to-day activities are not limited		
Access to vehicle	No cars or vans in household	Living environment domain	Short-term adaptation sub-domain
Living alone	1 person household		
Tenure	Social rented: Rents from council or Local Authority		Longer-term adaptation sub-domain
	Social rented: Other social rented		
	Private rented: Private landlord or letting agency		
	Private rented: Other private rented		
Housing in poor condition – 2019 IMD	Modelled estimate of proportion of social and private homes that fail to meet Decent Homes standard		Housing condition sub-domain
Sex	% female	Separate metric	
Population density	Residents per km	Separate metric	

Heat and flooding risk were prioritised in this feasibility study due to local council feedback on need, informed by the challenges faced in supporting their population through recent summer heatwaves, episodes of severe flooding, and the projected future increases in heat hazard. Flooding data under historic and future scenarios were provided by Fathom, a UK-based flood hazard intelligence company [24]. A dataset was created of a 1 in 100-year hazard at a Representative Concentration Pathway (RCP) 8.5 scenario in 2050, representing a ‘typical’ flood preparation scenario, and a ‘disaster’ scenario using 1 in 1000-year hazard modelling with the same projections. These scenarios are equivalent to those used by the UK Environment Agency for flood hazard zoning [25], but with a higher spatial resolution of 10m and also adjusted for climate projections, with RCP 8.5 used. A detailed description of the flood model and its validation is provided by Bates et al. [24]. Lower Layer Super Output Areas (LSOAs), UK statistical geographies covering 1,000–3,000 people each, were ranked as deciles by the aggregated proportion of buildings that experience any coastal, fluvial, or pluvial flooding greater than 10 cm, anywhere in their footprint. Heat data was extracted from the Met Office's HadUK-Grid dataset at 1 km horizontal spatial resolution [26]. This historical data was used due to improved resolution above UK Climate Projections (UKCP18) and as it fulfilled the requirements for the feasibility nature of this study. Datasets were created for the June, July, and August 2015–2019 average maximum temperature to represent average summer daytime temperatures, as well as the proportion of days where the average temperature exceeded 15 °C, the mean temperature above which mortality risks in Southwest England increase, based on a temperature-mortality model derived from the 2005–2014 summer observations for the region [27]. In each case, LSOAs were ranked as deciles by the weighted average of these metrics, weighted according to the density of buildings within each LSOA.

The POC application was developed using an ArcGIS Enterprise platform. The vulnerability index, domain and sub-domain data attributes were linked to 2021 LSOA boundaries by LSOA code and uploaded to ArcGIS Enterprise as a single geospatial dataset. A web map was then created with geospatial views of the vulnerability indices and socio-demographic, health, and housing domains.

## 3 Ethics

The study did not require formal ethical approval as advised by the UKHSA research and public health practice ethics and governance group as no patient identifiable data was utilised.

## 4 Results

### 4.1 Stakeholder engagement

#### 4.1.1 Summary, local priorities, and levers for action

Themes on what was required of a mapping tool were gathered from 12 1-to-1 interviewed stakeholders and four small group workshops. Stakeholders reported a current lack of data on vulnerability and climate hazard exposure to help guide adaptation efforts. The highest priority described was the need to improve understanding where greatest vulnerability and exposure converge. Three key themes of needs were identified.

#### 4.1.2 Theme one: Engaging and communicating with communities

Stakeholders highlighted the requirement for close working with communities in building resilience to the effects of a changing climate. Easily accessible data that can be visualized could strengthen bonds with community groups and create a more enabling environment for action, by improving the understanding around what threats to prepare for, and where need may be greatest.

#### 4.1.3 Theme two: Reducing health inequalities

A priority for stakeholders was the reduction of health inequalities. There is currently sparse data on how climate change impacts differentially on UK populations. Those interviewed felt that the ability to direct attention to areas of greatest need could stimulate understanding of differential need. It was highlighted that adaptations can be dovetailed with other priorities, strengthening the business case for health equity climate change interventions.

#### 4.1.4 Theme three: Building adaptive capacity and planning for the future

Stakeholders reported a siloed experience of some efforts, where greater collaboration would have been preferred; a shared tool could stimulate partnership working. A key barrier to action identified was budget pressures; increased data may allow better prioritisation.

#### 4.1.5 Potential use cases

Potential use cases for the tool within the confines of flooding and heat exposure were explored with stakeholders. Fig. 3 presents described actions that a LA may have a role in providing or in supporting the provision of through partners, complementing actions by national bodies driven by their legal responsibilities. Although not exhaustive, it gives indication of the breadth of levers a LA may have, through which improved data may facilitate better understanding of need, access, or success of intervention.

For flooding and heatwave resilience, the tool was hoped to increase understanding of current and future population risk, allowing more targeted education, and building a stronger and more proactive knowledge base in the community. A primary use may be in targeting interventions, such as prioritising communications aiming to encourage sign up to early warning systems in the most at-risk groups. Understanding variation in risk may also allow better resource allocation such as renewing focus on ‘medium risk’ areas to build resilience early. By bringing public health intelligence together, the tool may facilitate engagement with wider partners in planning interventions and in contributions to climate action and local council plans.

In building resilience against flooding, the mapping tool was developed with the aim to increase understanding of vulnerable populations and their exposure risks, and support developments of more granular local plans. It may inform decision making on council-led infrastructure such as flood defences by directing to areas of greatest need, and in better engagement with communities by allowing better articulation of projected needs. Improved public health intelligence can add value in community resilience building, supporting community groups and safe haven planning, with planning for safe sites a key council priority. In longer term planning, the tool may facilitate increasing use of tailored adaptive pathways and inform spending considerations including sustainable drainage solutions, blue-green infrastructure, and support for special populations such as care homes and prisons by directing attention to areas of highest risk overlap.

For heatwave risk, access to increased public health intelligence through the tool could support planning decisions on infrastructure such as access to cool spaces and facilities and urban planting. Building supportive healthcare capacity and resilience was considered key, with the tool enabling decision making on training health visitors and community outreach workers and supporting better collaboration with healthcare providers as potential service demands can be better appreciated. A role was seen in long-term spending reviews such as on planting and green space to reflect heat in urban areas. Some examples had additional benefits such as green space access links to mental and physical health improvement, which was felt to promote a reduction in siloed working. Many interventions could be dovetailed with other local priorities to meet an array of objectives outside of the climate adaptation imperative.

### 4.2 Building a mapping tool

#### 4.2.1 Data on health equity and inclusion health groups

Data on inclusion health groups as considered by the CORE20PLUS framework [9] are often not consistently captured. SGC holds additional, local data; however, potential for national rollout of the tool precluded central hosting of local, potentially identifiable data.

#### 4.2.2 Measures of vulnerability and hazard

**Fig. 2:**
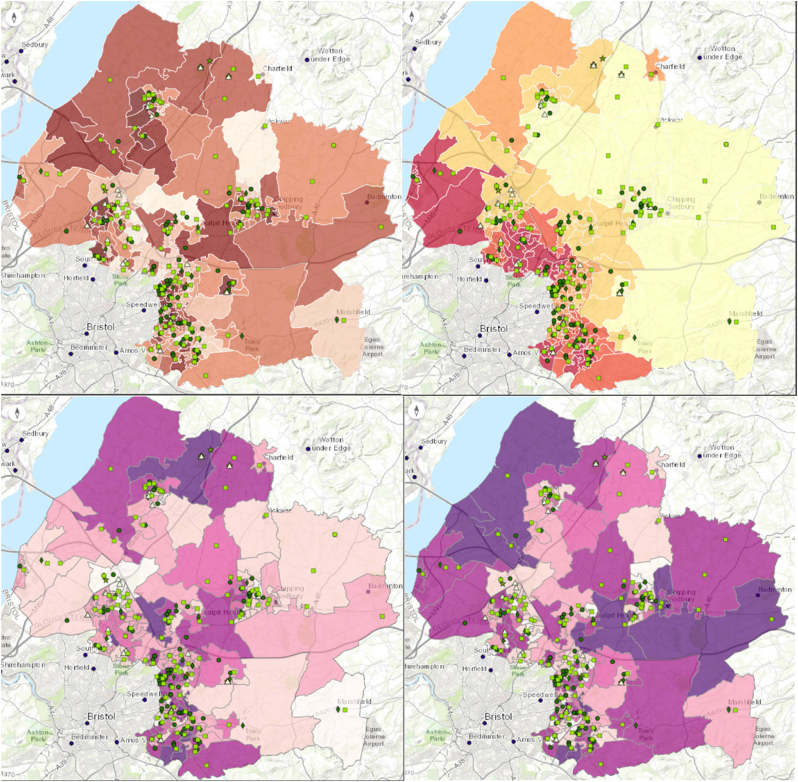
Proof-of-concept application screenshots (presented only for display purposes) with example LSOAs highlighted. These are given only as examples of the tool developed.

An index for measuring vulnerability was created [supplementary material] including 33 metrics, largely from census data, combined into 9 sub domains contributing to four domains of adaptive capacity, health, sensitivity, and living environment [Table 1]. LSOA scores for vulnerability and exposure were split into terciles and plotted onto a 3x3 risk matrix for visualisation. Vulnerability and heat exposure risk matrix score was mapped for the ‘heat vulnerability’ layer, and vulnerability and flooding risk matrix score for the ‘flooding vulnerability’ layer [Fig. 2].

**Fig. 3:**
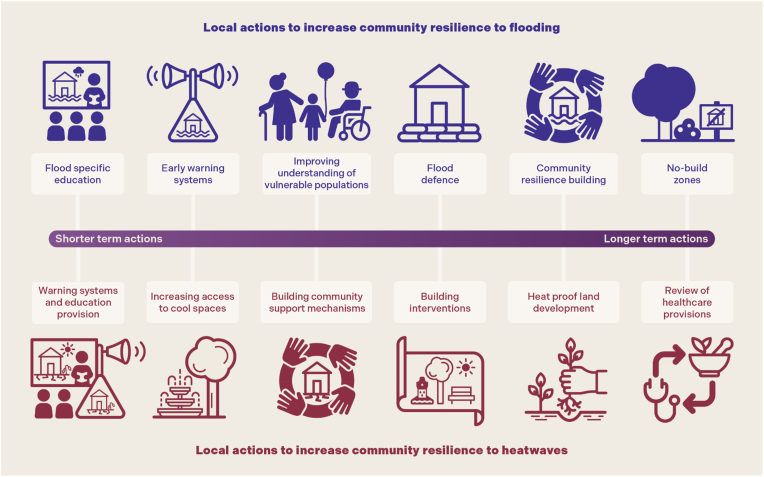
Stakeholder engagement facilitated the working group's understanding of the type and breadth of interventions to increase community resilience to heat and flooding that a LA may have a role in providing or in supporting the provision of through partners, some of which are presented here.

## 5 Discussion

Local Authorities have significant potential influence and levers for action to increase community resilience to the health impacts of climate-related hazards. However, our stakeholder engagement suggested key barriers to action in resource constraints, siloed nature of some working, and a lack of public health intelligence to provide an evidence base for decision making. Resource constraints have been noted nationally as the main barrier to climate adaptation [28].

Improved public health intelligence can help create a more enabling environment to resilience building by informing decision making processes [15]. We aimed to understand through this feasibility study where increased public health intelligence in the form of bringing together sociodemographic vulnerability and exposure risk data may add value to current LA processes. Demand for this was noted by engaged stakeholders, with key needs highlighted in engaging communities and partners better, informing decision making including in resource allocation, reducing inequalities, and targeting planning and interventions to where future need may be greatest. This conforms with available evidence [16,17], but awareness of the issues may not alone assure impact of developed interventions [18,19]. Whilst other similar tools have been created, these are not always co-developed with LA stakeholders who may be a key target for future utilisation of such tools [18]. By design of a novel mapping tool, created with early co-development with the intended users, we hope to produce a tool that will have acceptability and impact. Whether this approach increases the utility of the tool will now be assessed in a pilot study with the same LA, which will look to further understand the ways it may be used above those suggested in Fig. 3.

To address the needs identified, we present a mapping tool informed by a newly created index of vulnerability and exposure data. Whilst measuring vulnerability is notoriously problematic due to the complexity involved, it was anticipated by stakeholders that the added ability to understand risk and exposure together may allow additional nuance in understanding and decision making. The potential uses of this tool were explored with the LA, facilitating deeper understanding of where additional public health intelligence may add value in planning, education and training, and community engagement processes. It is acknowledged that many factors such as budgetary pressures and lack of clear use case may limit uptake; the subsequent pilot study has been planned to gather further information on how, where, and why such a tool may be used. Depending on the results of this pilot, next steps may be to consider in which contexts the tool holds most value or whether it could be used more widely, in other areas. By promoting increased collaborative working, shared mapping tools may facilitate better appreciation of wider and co-benefits to adaptation interventions, and where synergies between health improvement efforts can be found, facilitating a reduction in siloed working, and enabling a tangible potential added value to encourage use of our tool. This should enable gains in health equity efforts, a key concern of SGC and LAs nationally [28], and enable progress towards a ‘just transition’, limiting unintended negative consequences on vulnerable groups [29,30].

Our feasibility study shows identified LA needs and adds evidence to the technical specification of the support required, enabled through better mobilisation of public health intelligence. We add value to current mapping tools by processing vulnerability and hazard data to show the overlap of risk to support understanding of this complex picture, helping local practitioners to consider wider determinants, drivers, and pathways. Additional value will come from planned piloting and evaluation of the mapping tool. Limitations in our work include that we engaged with a single local authority to co-develop this tool; perceptions and priorities at other LAs may vary. Within engaged stakeholders, there was limited clarity on how this tool may be applied operationally: explicit examples of need remained difficult to define. This may be clear in the expressed ‘hope’ that the tool may be used in the suggested ways. Whether this occurs will be assessed in subsequent work. Whilst our feasibility study develops a proposal for a tool and guided the development of ours, we have not yet evaluated this in practice or tested its acceptability or applicability. The measurement of vulnerability is limited by the contained data, including a lack of information on some key populations including inclusion health groups. Measuring vulnerability necessitates ignoring ethnographic understanding of complex variables such as resilience, social capital, and behavioural norms, which are likely key to any community's ability to adapt and respond. This tool is intended only to support local understanding, not as a primary or complete information resource.

## 6 Conclusions

This feasibility study allowed deeper understanding of LA needs and potential use cases for mapping tools of vulnerability to climate related hazards and supports the development of a technical specification for subsequent iterations. The tool developed should now be trialled and evaluated, to understand further its applicability and acceptability to increase learning about how LA efforts to increase resilience can be supported in this way. Uptake will be explored of the voluntary use, and feedback when used, to see where the tool is seen to add value, how it informs decision making, and if changes might be required to improve on this and increase applicability to other geographic areas. This might include capacity to bring in additional vulnerability data, consideration of different hazards, or more directive approach to its use in more limited use cases. Co-development of the tool throughout the process facilitated the understanding of where support was required. However, it is notable that the potential uses described cover only an aspect of the large system of population resilience, for instance neglecting health service interventions. Whilst the tool was felt to potentially be a useful instrument to supplement practice, broader context could have been gained by engaging with more stakeholders, including those representing wider partners such as integrated care boards or local resilience forums. Provision of additional public health intelligence through tools such as this is likely to be beneficial to the support and guidance LAs need; more work is required in understanding how best to achieve this.

## Funding

Y.T.E.L. was funded by the University of Bristol Climate Change and Health Fellowship. JH holds an NIHR Academic Clinical Fellowship (ACF-2022-13-013).

## Data availability

The source code used for the data analysis is available at: https://github.com/JGIBristol/mapping-vulnerability-climate-hazards [31].

The data underlying the analysis is available as follows: The heat data is part of the HadUK-Grid dataset available from the Met Office (ref Met Office et al., 2022). The flooding data was kindly provided by Fathom (ref. Bates, P.D. et al., 2023) under licence. The vulnerability data is part of the England and Wales Census 2021 and English indices of deprivation datasets available from the 10.13039/100018517Office for National Statistics. Supporting geospatial data is available from 10.13039/100012721Ordnance Survey and 10.13039/100018517Office for National Statistics. More details are provided in the source code.

## Declaration of competing interest

The authors declare that they have no known competing financial interests or personal relationships that could have appeared to influence the work reported in this paper.
