# Supplementary material for: Mapping vulnerability to climate-related hazards to inform local authority action in adaptation: A feasibility study
Source: Public Health Pract (Oxf). 2024 Sep 21;8:100549. doi: 10.1016/j.puhip.2024.100549 (PMC11472114; doi:10.1016/j.puhip.2024.100549)
Supplement: Multimedia component 1 [file mmc1.docx]

**Supplementary material**

**Table 2** Local authority engagement map showing stakeholders engaged in discussions

| **Type of discussion** | **Organisation** | **Staff or group engaged with** |
| --- | --- | --- |
| Individual and small group discussions | South Gloucestershire Council | Climate Emergency Manager |
|  |  | Senior Environmental Change Officer |
|  |  | Public Health Strategic Lead – Health Protection |
|  |  | Consultant in Public Health |
|  |  | Public Health Strategic Lead – Evidence, Population Health, and Intelligence |
|  |  | Public Health Strategic Lead for Built Environments |
|  | South Gloucestershire Locality Partnership and Integrated Care Board | Locality Partnership Manager |
|  |  | Head of Population Health management |
|  |  | Community Agent |
|  | VCSE (one a local care provider and one a community partnership) | Chief Executive |
|  |  | Executive lead for climate and sustainability |
|  |  | Associate director for estates and environment |
| Group presentations, workshops | South Gloucestershire Council | Health Protection Assurance Group |
|  |  | Ageing well core group |
|  |  | Open workshop for staff |
|  |  | Senior Leadership Team |

**Vulnerability index methodology**

Vulnerability measurement through index aggregation followed the hierarchical methodology described by Tate (22) and used in the English Indices of Multiple Deprivation (IMD) (23). Metrics were selected from the literature, considered for inclusion by expert panel, and translated into data available reliably at Lower Layer Super Output Area (LSOA) level. Predominantly census data was used, however if no census-derived proxy was available other sources such as the IMD were considered. If data was not published down to LSOA level, then data from the next administrative level, Middle Layer Super Output Area (MSOA), was used instead.

The metrics were then analysed for inclusion into the index, with data analysis described below, and grouped into domains and sub-domains [table 2]. This is in line with CCRA identification and is mirrored nationally with most LAs citing heat and flooding as their most significant hazards (29).

The numerical stages of the methodology were:

**Combination of individual metrics**Individual metrics measuring different aspects or facets of the same underlying element of vulnerability were first added together if this could be done without overlap or double counting. For example, in the education metric [table 2], the categories “No qualifications”, “Level 1” and “Level 2” could simply be added as there is no overlap between them. In the case of the income metric from IMD data, this summation had already been performed. Shrinkage was not applied.

**Transformation** The majority of combined metrics were counts of people or households in each LSOA. To allow LSOAs to be compared fairly, these were converted to percentages using an appropriate denominator that related to the total number of people or households ‘at risk’. For example, for “No cars or vans in household” the appropriate denominator was the total number of households in the LSOA, whereas for the education metric it was the number of residents aged 16 years and over. The only metric that was not converted to a percentage was income, which was provided as a rank.

**Normalisation** For each sub-domain (or in the case of health, domain) the remaining metrics were then placed on a common and dimensionless scale by ranking and transforming to a standard normal distribution based on their ranks.

**Weighting and aggregation** The normalised metrics were then combined to produce sub-domain scores (or in the case of health, domain scores). As no sub-domain had more than two constituent metrics, a factor analysis was not used to determine weightings and the metrics were simply summed using a weight of +1 or −1 such that larger scores could be attributed to higher vulnerability.

The sub-domain scores were then converted to a truncated exponential distribution according to (25, appendix F) by ranking and then using the equation:

$$X=-23\ln\left( 1-R\left( 1-{exp}^{-\frac{100}{23}} \right) \right)$$

where *X* is the converted sub-domain score and *R* is the rank, scaled to the range (0, 1]. The conversion to a truncated exponential was performed so that if an LSOA scored highly in one sub-domain (or domain), this could not be cancelled out by a low score in another. As such, vulnerability in one sub-domain generally rendered the LSOA as vulnerable overall. The sub-domains were summed with equal weighting to yield the domain scores.

The domain scores were again converted to a truncated exponential distribution to reduce cancellation effects. The domain scores were than summed with equal weighting (in the absence of a strong justification to apply weighting) to yield the overall vulnerability index, which was ranked and converted to deciles.

**Index aggregation**

The top-level domains are:

- Adaptive capacity,
- Health,
- Sensitivity,
- Living environment.

The sub-domains are:

- Adaptive capacity: income,
- Adaptive capacity: language,
- Adaptive capacity: helping others,
- Sensitivity: younger people,
- Sensitivity: older, people
- Living environment short term adaptation,
- Living environment longer term adaptation,
- Living environment condition

[health does not have any sub-domains]

**Table 3** Initial metrics considered from literature with explanation of working group assessment

| Broad theme | Metric | Suitability for inclusion decision by group | |
| --- | --- | --- | --- |
| Socio-demographic | Age % >65 or <5 | Included | Different upper limit options in literature. >65 years old chosen by working group |
|  | Gender | Included | Available from census |
|  | Married | Not included | Living alone felt a better marker (included below) |
|  | Ethnicity - non-English speaking or English not first language or % migrant population in area | Not included | Census gives English capability |
|  | Education: > 12 years education | Included | Up to GCSE equivalent taken from Census |
|  | % unemployed | Included | Combined and captured by IMD income domain |
|  | Welfare (universal credit) recipients | Included |  |
|  | Income brackets | Included |  |
|  | % living in poverty | Included |  |
|  | Recipients of free school meals | Included |  |
|  | Median home value | Not included | Vulnerability proxy better covered by above IMD income |
|  | Tenure (renting) | Included | Available from census |
|  | Access to vehicles (no car) | Included | Available from census |
|  | % carers | Included | Available from census |
|  | % employed in farming | Not included | Not translatable - too specific |
| Health | % very bad or very good health | Included | Available from census |
|  | % with disability or long term health condition | Included | Available from census |
|  | Medication use | Not included | Poor relation to outcomes, 'health' better proxy |
|  | Medical condition / co-morbidities | Not included | Perceived better covered individually once data linkage available |
|  | % population in care homes | Not included | Not well available at LSOA level. Consider point mapping care homes instead |
|  | IMD health domain | Not included | Dropped in favour of other health metrics (otherwise double counted), older data |
| Housing / living environment | % without central heating | Not included | Only relevant for cold |
|  | % housing before 1945 or Year property built | Included | Adapted for availability to census - IMD Housing in poor condition |
|  | % mobile home | Not included | Available from census but low variance – very low numbers. |
|  | % private water supply | Not included | Unclear proxy for all-hazard vulnerability |
|  | Urban vs rural marker | Not included | Discussed with group - population density simplest so carried forward. Could consider specific urban / rural marker in future iteration |
|  | Population density | Included |  |
|  | % Vacant houses | Not included | Rarely relevant in UK and complex for example rental market vs under-developed |
|  | Accommodation type | Not included | LSOA data is poorly distributed (small numbers) |
| Health infrastructure | Number of people per GP | Not included | Rarely included elsewhere. Likely poor proxy for vulnerability and unlikely target for local authority. Key infrastructure data could instead be given as point mapping such as location of care homes / hospitals. |
|  | Number of hospital beds (per 1,000) |  |  |
|  | Road distance to: GP surgery / supermarket |  |  |
| Prior exposure | Previously flooded | Not included | Previous exposure less relevant if adaptation carried out. Better captured in hazard section |
